# A high-throughput HPLC method for simultaneous quantification of pyrethroid and pyriproxyfen in long-lasting insecticide-treated nets

**DOI:** 10.1038/s41598-022-13768-z

**Published:** 2022-06-11

**Authors:** Kyle J. Walker, Christopher T. Williams, Folasade O. Oladepo, John Lucas, David Malone, Mark J. I. Paine, Hanafy M. Ismail

**Affiliations:** 1 Vector Biology Department, Liverpool School of Tropical Medicine, Pembroke Pl, Liverpool, L3 5QA UK; 2 Cowleigh Park Farm, Cowleigh Road, Malvern, WR13 5HJ UK; 3 Innovative Vector Control Consortium, Liverpool School of Tropical Medicine, Pembroke Place, Liverpool, L3 5QA UK

**Keywords:** Analytical chemistry, Malaria, Polymers

## Abstract

Long-lasting insecticide-treated nets (LLINs) play a crucial role in preventing malaria transmission. LLINs should remain effective for at least three years, even after repeated washings. Currently, monitoring insecticides in LLINs is cumbersome, costly, and requires specialized equipment and hazardous solvents. Our aim was to develop a simple, high-throughput and low-resource method for measuring insecticides in LLINs. To extract insecticides, polyethylene-LLIN samples were heated at 85 °C for 45 min in a non-hazardous solvent mix containing dicyclohexylphthalate as an internal standard. The extraction solvent was reduced from 50 to 5 ml using a 0.2 g sample, 90% smaller than the recommended sample size. By optimizing HPLC chromatography, we simultaneously detected pyrethroid and pyriproxyfen insecticides with high sensitivity in LLIN's extract. The method can quantify levels ≥ 0.0015% permethrin, 0.00045% alpha-cypermethrin and 0.00025% pyriproxyfen (w/w) in polyethylene, allowing for insecticide tracking before and after the use of LLINs. This method can be used to assess LLINs with 1% pyriproxyfen (pyriproxyfen-LLIN) or 2% permethrin (Olyset® Net), 1% pyriproxyfen and 2% permethrin (Olyset® Duo), or 0.55% pyriproxyfen and 0.55% alpha-cypermethrin (Royal Gaurd®). One can run 120 samples (40 nets) simultaneously with high precision and accuracy, improving throughput and reducing labour, costs, and environmental impact.

## Introduction

Human deaths due to malaria declined by approximately 50% between 2000 and 2015^1,2^, primarily due to the development, scale-up and universal distribution of long-lasting insecticide-treated nets (LLINs)^1^. Nearly 2.2 billion insecticide treated nets have been delivered worldwide since 2004, of which 1.9 billion (86%) were supplied to Sub-Saharan Africa^3^ preventing up to 68% of the malaria cases in the region^2^. LLINs reduce malaria transmission by acting as a physical barrier to block mosquito-human contact and killing and repelling mosquitoes by the insecticide^3,4^.

**Figure 1:**
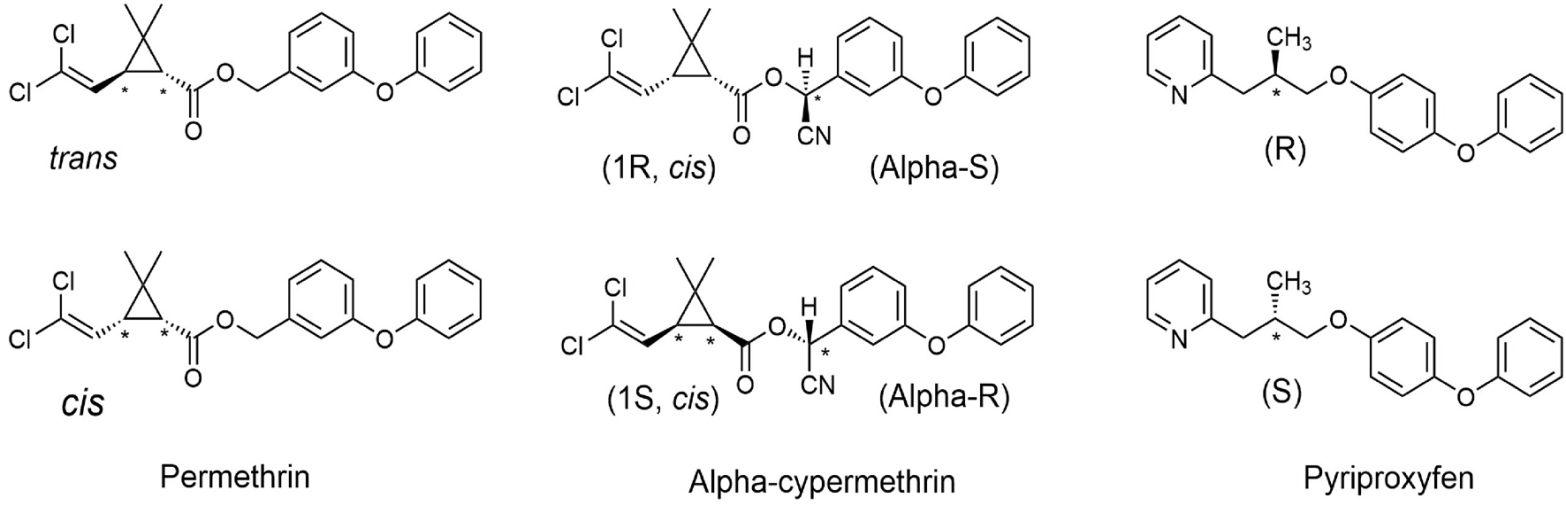
Chemical structure of permethrin, alpha-cypermethrin and pyriproxyfen insecticides (*: chiral centres).

The World Health Organization (WHO) recommends using pyrethroids (Fig. 1) in LLINs, as they are highly toxic to mosquitoes, but not to mammals^3,4^. However, since 2016, there have been worrying signs of malaria resurgence in many areas of Sub-Saharan Africa, primarily due to the rapid evolution of pyrethroid resistance in mosquitoes^3^. In light of the impact of pyrethroid resistance on malaria control, dual-action LLINs are being developed to delay the development of resistance and extend the lifespan of both active ingredients^5–9^. Royal Guard® Net for instance was prequalified by WHO in March 2019 and has shown enhanced efficiency against *Anopheles gambiae* mosquitoes before and after 20 standardised washes in laboratory and experimental hut trials^10^.

**Figure 2:**
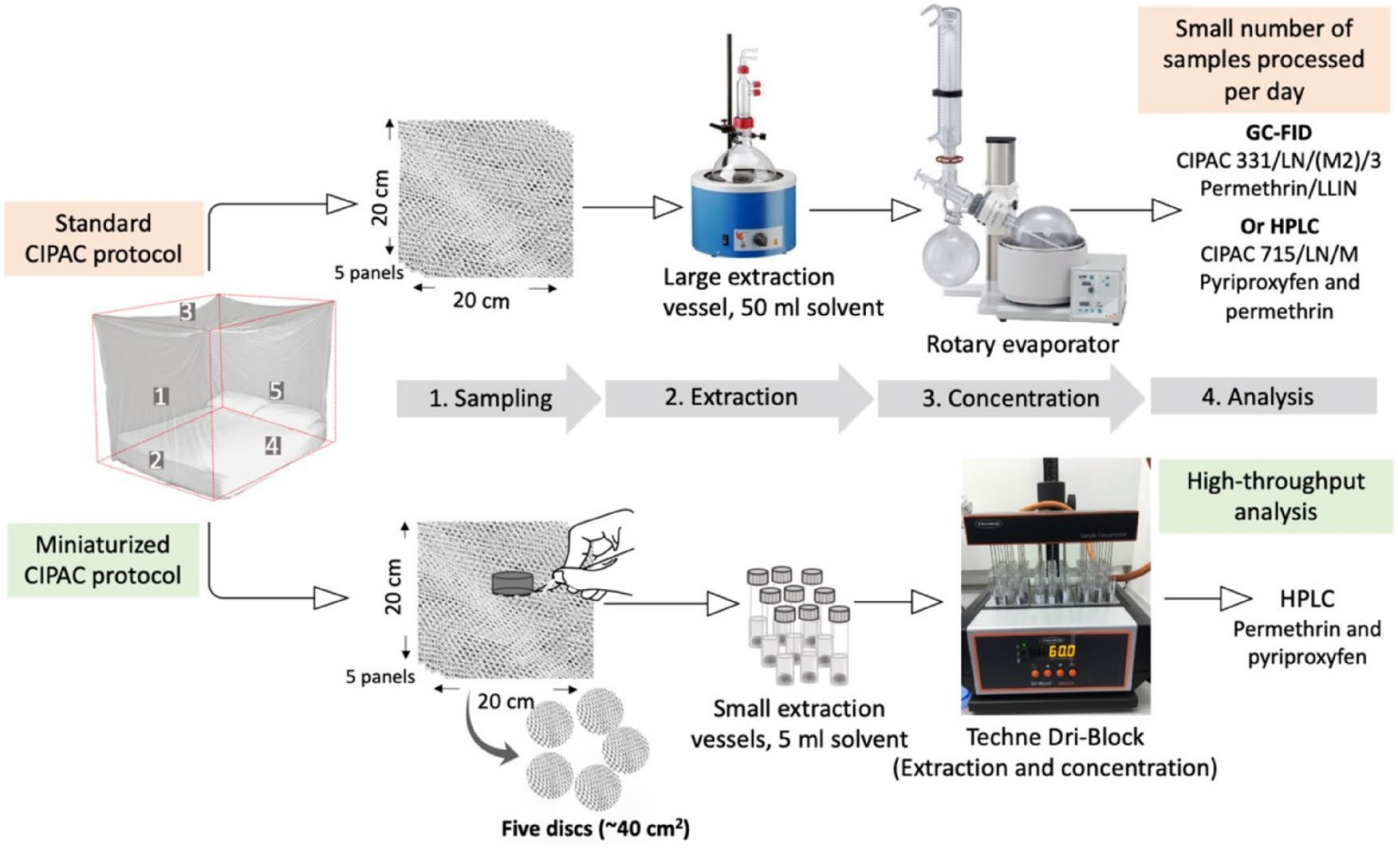
Comparison of standard CIPAC method with a miniaturised protocol for determining insecticide content incorporated in long-lasting bed nets (LLINs). The sample size has been reduced from 400 cm^2^ (2 g) to ~ 40 cm^2^ (0.2 g) to enable a small volume of extraction solution (5 vs. 50 used in the standard CIPAC methods) for permethrin^12^ and pyriproxyfen^13^ respectively.

However, new nets must adhere to the guidelines from the WHO Prequalification Team for Vector Control Products (PQT-VC) in relation to insecticide content, wash resistance, storage stability, bio-efficacy, and field trials^11^. This requires the parallel development of analytical approaches for new product quality control assessment (QCA). Also, given the imminent arrival of new LLINs into the vector control market, the development of ‘accessible’ methods for quantifying insecticides will be necessary for stakeholders such as procurement agencies and vector control operatives to monitor the quality of the bed nets being used for malaria control operations. Standard Collaborative International Pesticides Analytical Council (CIPAC) methods that utilize chromatographic techniques are available for insecticide quantification^12,13^ and referenced in WHO testing specifications for LLINs^11^. For instance, the standard CIPAC protocol for analyzing pyriproxyfen content in LLIN (715/LN/M, CIPAC Handbook O, page 143) is suitable for determining pyriproxyfen content in nets containing pyriproxyfen as the only active ingredient and in mixtures with permethrin^13^. Also, the HPLC method for pyrethroid quantification has been developed to provide a universal protocol for detecting and analyzing pyrethroids from both coated and incorporated nets^14^. But currently, there is no universal HPLC method available for simultaneous quantification of dual active ingredients, such as pyrethroid and pyriproxyfen. Moreover, all available methods rely on a large sample size (~ 2 g of net mass equivalent to ~ 400 cm^2^), consume large volumes of organic solvents that require large extraction vessels and use a rotary evaporator for sample concentration (Fig. 2). Contrary to the aims of green chemistry, there are potential adverse effects to the environment resulting from large volume solvent consumption^15^. Furthermore, these methods are labour-intensive, time-consuming and costly, providing barriers to their being implemented in country for routine QCA.

Here we have modified the sampling method of LLINs to reduce the sample size of LLIN and the consumption of organic solvent to simplify the extraction and quantification procedure for insecticide(s) in LLINs. In addition, we have optimized the chromatographic conditions used in the standard CIPAC protocol for QCA of pyriproxyfen-LLIN^13^ to improve the HPLC sensitivity for pyrethroid quantification alone or in combination with pyriproxyfen. A range of prototype and commercial LLINs, *i.e.* Pyriproxyfen-Net (Pyriproxyfen), Olyset® Net (Permethrin), Olyset® Duo (permethrin and pyriproxyfen mixture) and Royal Guard® (alpha-cypermethrin and pyriproxyfen mixture) were used to assess the optimized method for insecticide(s) quantification specificity, accuracy, precession, and reproducibility. Results indicate that the new method is suitable for quantifying insecticide(s) content in LLINs containing pyriproxyfen and/or pyrethroid active ingredient. The new method provides high throughput analytical capacity for insecticide(s) quantification in LLINs.

## Methods

### Reagents

Technical grade insecticide standards for HPLC analysis were obtained from Sigma Aldrich—permethrin 98.3% purity (57.8% *trans*-isomer, 40.5% *cis*-isomer); alpha-cypermethrin, ≥ 98% purity). HPLC grade acetonitrile (≥ 99%), water and heptane were obtained from Fisher Chemicals. 1-propanol (≥ 99%) was obtained from Across Organics. Four types of LLIN were obtained from different suppliers (Table 1).

**Table 1:** Manufacturer and insecticide information for LLINs.

LLIN Name	Manufacturer	Denier	Material	Active ingredient concentration
Pyriproxyfen-Net	Sumitomo Chemical (Japan)	150	Polyethylene	Pyriproxyfen (10 g/Kg)
Olyset® Net	Sumitomo Chemical (Japan)	150	Polyethylene	Permethrin (20 g/Kg)
Olyset® Duo	Sumitomo Chemical (Japan)	150	Polyethylene	Permethrin (20 g/Kg) + Pyriproxyfen (10 g/Kg)
Royal Guard®	Disease Control Technologies, LLC (USA)	120	Polyethylene	Alpha-cypermethrin (5.5 g/Kg) + Pyriproxyfen (5.5 g/Kg)

HPLC analysis was performed with a Dionex UltiMate 3000 comprising an autosampler (WPS 3000 SL), quaternary pump (LPG 3400 SD), and variable wavelength detector (VWP 3410 RS). Peak areas were obtained using Chromeleon software (Chromeleon 7.2 SR4). The column used was a Hypersil GOLD C18 column (75 Å, 250 × 4.6 mm, 5-μm particle size; Thermo Scientific). Peak purity analysis was carried out using a Thermo Fisher Scientific Vanquish Core HPLC System comprised of a Vanquish™ Split Sampler (VC-A12-A), Vanquish™ Column Compartment (VC-C10-A), Vanquish™ Binary Pump (VC-P10-A), and Vanquish™ Diode Array Detector; multiple wavelength detector (VC-D11-A).

### Optimized test method summary

The method below outlines a single analysis of a single net. The methods for the validation experiments are outlined in later experimental sections. Whole nets consisting of five panels were tested. A small square (approximately 25 × 25 cm^2^) was cut from each to perform a representative analysis of the whole net. These are laid on top of each other, and a small disc (~ 8 cm^2^) cut from each using a stencil and disposable scalpel. The total weight of the five discs was recorded before transferring to the 10 ml extraction tube (Wheaton® 10 ml soda-lime glass with polypropylene cap). Five millilitres of the extraction solution of 10% 1-propanol in heptane containing 100 µg/dicyclohexyl phthalate [DCP] as an internal extraction control was added, ensuring all the net were submerged in the solution. The glass tubes were capped with tin foil and sealed with screw lid to prevent solvent loss, following by heating at 85 °C for 45 min using a Dri-Block® (Techne) heater in a fume hood. One milliliter was then transferred to a new glass tube and evaporated at 60 °C under compressed air in a fume hood, then resuspended in 1 ml acetonitrile and vortexed for one minute at 2500–3000 rpm before decanting into a 1.5 microcentrifuge tube. The sample was filtered through a PTFE 0.2 µm filter before transferring 100 µl to an HPLC vial for analysis. Standards of concentrations (31.25 µg/, 62.5 µg/, 125 µg/, 250 µg/, 500 µg/) were prepared for each insecticide present in the nets being analysed. The HPLC method incorporated an isocratic mobile phase of 70% acetonitrile and 30% water, a 1/min flow rate, 40-min run time and an analysis wavelength of 226 nm. The quantities of permethrin and pyriproxyfen in g/kg are calculated from standard curves produced from the known standard concentrations and corrected against the internal DCP controls. The final insecticide content in g/kg was estimated using the following equation:

$$I = \left( \frac{x}{a} \right) \times \left( {\frac{0.001}{m}} \right) \times C \times {\text{f}}$$

where *I* is the insecticide content in g/kg, and *x* is the insecticide peak area at 226 nm, (for permethrin the *cis-* and *trans-* isomer peak areas were combined). *a* is the slope of the relevant insecticide standard curve. *m* is the mass of the net sample. *C* is the internal standard correction factor, calculated by dividing the average peak area of DCP controls by the DCP peak area obtained for the sample. *f* is the sample dilution factor.

### Specificity

To check the method specificity, chromatogram peaks of extraction solutions from Olyset® Duo® and Royal Guard® were compared with that of analytical grade insecticides (permethrin and pyriproxyfen). We confirmed there was no overlap of the insecticide peaks with either the internal control DCP or contamainants peaks co-extracted from polyethylene matrix. The chromatograms produced from these samples were also analyzed for any obvious peak shouldering, tailing or crossover. The insecticide peak retention time was also compared to that of the injected standards, and the percentage retention time was calculated from the following formula:

$$\% {\text{RT }} = RT_{sample} /RT_{standard} \times 100$$

### Linearity

Linear regression analysis was used to validate the linearity of HPLC for quantification of five working standard solutions of permethrin, alpha-cypermethrin and pyriproxyfen. The standards used ranged from 31.25 to 500 µg/ as produced during the net analysis. The average peak area, standard deviation, and relative standard deviation (%RSD) were recorded for each insecticide concentration. By injecting 20 µl of insecticide concentrations 31.25, 62.5, 125, 250 and 500 µg/, the response should be linear with R^2^ > 0.9. The linearity was evaluated by generating the calibration curves presented by the following linear regression analysis equation:

1 $$y = ax + b$$

The linearity was obtained by plotting the peak areas (y, mAU) of insecticide versus injected standard concentration (µg/) onto a column and by the value of their correlation coefficients (R^2^). For each of the three standard curves produced, the slope value is recorded. The average slope (a), standard deviation (σ) and %RSD of these slopes are also reported.

### Limit of detection (LoD) and limit of quantification (LoQ)

LoD and LoQ assays were performed for both insecticides. According to the HPLC conditions described above, a 20 µl of standard curve ranging from 0.007 to 250 µg/ was injected in triplicate. The LoD and LoQ were calculated by regression analysis slope (a) obtained from “Eq. (1)” and the standard deviation (σ) value of the line obtained by analyzing these low-concentration solutions and following equations:

2 $$LoD \, = \, 3.3\sigma /a$$

3 $$LoQ \, = 10\sigma /a$$

### Insecticide recovery

A recovery experiment was conducted to confirm that insecticides content was determined accurately with high precision. The samples subjected to this assessment were untreated nets fortified with concentrations of permethrin and pyriproxyfen at the specification level for each insecticide. Four nets were analyzed per concentration. The results were analyzed, and the following equation was used for the recoveries of the insecticides calculations:

$$R = \frac{C}{Cs} \times 100$$

where R recovery %, C: observed concentration of the insecticide (µg/) and Cs: fortified concentration (µg/) permethrin.

### Heat stability

A comparative assay was performed to assess the stability of the insecticides when heated to 85 °C for 45 min, comparing results with and without heating. For the heat stability experiment, 5 of insecticide at two concentrations, 0.4 and 0.2 mg/(w/v) in extraction solution were heated in triplicate at 85 °C for 45 min. 1 of the solution was removed, evaporated, and reconstituted in 1 of HPLC-grade acetonitrile for HPLC analysis. In parallel, 1 unheated samples from the insecticide standard were evaporated and reconstituted in 1 acetonitrile to compare HPLC chromatograms of heated versus unheated treatments. All samples were then treated the same as described in the test method. The average insecticide recovered, standard deviation and %RSD for heating and non-heating methods were reported for each insecticide.

### Quality control assessment of polyethylene-based LLIN formulations

To evaluate the suitability of the optimized method to analyze LLINs incorporating pyriproxyfen and/or pyrethroids, Prototype pyriproxyfen LLIN, Olyset®, Olyset® Duo and Royal Guard® nets (Table 1) were analyzed with the optimized method.

### Accuracy and precision

Twenty-four new nets from Olyset® and Olyset® Duo (Table 1) were analyzed in triplicate as part of accuracy and precision studies. Precision was measured by relative standard deviation (%RSD). The accuracy was calculated using the formula (mean concentration found/target concentration) × 100. For accuracy, the data had to fall within the range of ± 25% of target manufacture dose. Precision of the developed method for Royal Guard® LLIN was evaluated on an intraday and interday basis. Assay precision (intraday precision) was calculated using %RSD for six replicates of the QC sample, and inter-day precision was determined based on the analysis of six replicates of the QC sample on three consecutive days.

## Results

### Improvement of HPLC analysis

**Figure 3:**
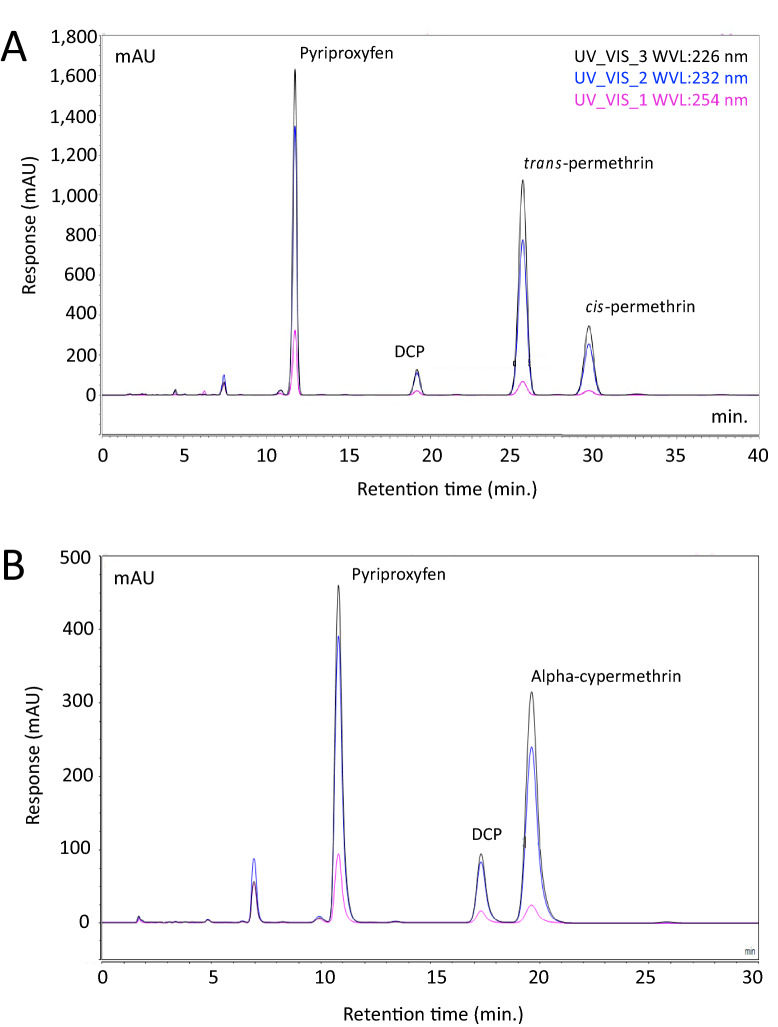
HPLC chromatogram for pyriproxyfen and pyrethroids extracted from Olyset® Duo and Royal Guard® LLINs with reference to internal standard ‘dicyclohexyl phthalate (DCP). (**A**) Olyset® Duo active ingredients, pyriproxyfen and *trans*-permethrin and *cis* permethrin, measured by HPLC-diode array detector (DAD) at three-wavelength 226 (black), 232 (blue) and 254 (purple) nm in LLIN extraction solution. (**B**) Royal Guard® active ingredients; pyriproxyfen, and alpha*-*cypermethrin, were detected at the same three-wavelength in the sample solution following LLIN extraction.

To increase the HPLC sensitivity for the simultaneous analysis of pyriproxyfen and pyrethroids in LLINs, we optimized the analytical chromatographic conditions in the standard CIPAC protocol recommended for quantifying pyriproxyfen in pyriproxyfen-LLIN^13^. Olyset® Duo LLIN manufactured with 20 g/kg permethrin (2% w/w) and 10 g/kg pyriproxyfen (1% w/w) and Royal Guard® LLIN manufactured with 5.5 g/kg alpha-cypermethrin (0.55%) and 5.5 g/kg pyriproxyfen (0.55%) were used as the test materials for HPLC method improvement. Extracts from ~ 0.2 g of LLIN were investigated for detection sensitivity using a Vanquish™ Diode Array Detector (VC-D11-A) at shorter wavelengths of 226 and 232 nm compared to the recommended wavelength of 254 nm^13^. The resulting chromatograms are presented in Fig. 3. All three insecticides produced the highest peak heights and corresponding peak areas at 226 nm (Fig. 3). At this wavelength, the greatest sensitivity was recorded for pyriproxyfen with LoD and LoQ of 0.04 µg/ (1 mg/kg net) and 0.1 µg/ (2.5 mg/kg net) respectively, followed by alpha-cypermethrin with LoD and LoQ of 0.06 µg/ (1.5 mg/kg) and 0.18 µg/ (4.5 mg/kg) respectively, and permethrin (*cis* and *trans*)) with LoD and LoQ of 2 µg/ (5 mg/kg net) and 0.6 µg/ (15 mg/kg net), respectively. DCP with a retention time well separated from the target insecticides was used as an internal standard to correct for volume errors and to ensure high reproducibility between samples. Four well-separated peaks of pyriproxyfen, DCP, *trans*-permethrin and *cis-*permethrin were obtained with Olyset® Duo sample (Fig. 3A), and three separat peaks, pyriproxyfen, DCP and alpha-cypermethrin were obtained with Royal Guard® sample (Fig. 3B). An ambient column temperature (23 °C) was also used to ensure the method suitability across different laboratory settings. At this temperature, the optimized acetonitrile/water mobile phase ratio 70:30 (v/v), which was slightly higher than the 66.6–33.3 (v/v) recommended method (CIPAC), produced symmetric analyte peaks with no sign of peak abnormalities and clear analyte separation (Fig. 3). Under these conditions the run times for Olyset® Duo and Royal Guard® were 40 min (Fig. 3A) and 30 min (Fig. 3B) respectively compared with 60 min per run in the standard CIPAC method^13^.

### Specificity

The improved method was also assessed for method sepecificity to test its ability to measure accurately and specifically the insecticide of interest in the presence of other components that may be coextracted from the net matrix. Therfore, insecticide peaks determined in both samples were further investigated for the presence of visible interferences (shoulders) by comparison with retention times from insecticide standard injections. Sample retention time of analytes matched the standards with calculated percentage retention times of 100.11% (pyriproxyfen), 100.1% (DCP), 100.23% (*trans*-permethrin), 100.22% (c*is*-permethrin) for sample extracted from Olyset® Duo (Fig. S1). Similarly, samples extracted from Royal Guard® Net exhibited 100.11% and 100.07% matching retention time for pyriproxyfen and alpha-cypermethrin, respectively (Fig. S2). In addition, the average peak purities for pyriproxyfen (997), *trans*-permethrin (1000) and *cis*-permethrin (1000) from sample solutions extracted from Olyset® Duo Net matched the pure analyte peak factor of 1000 (Fig. S1) and for pyriproxyfen (998) and alpha-cypermethrin (1000) extracted from Royal Guard® Net (Fig. S2).

### Linearity

The linearity of the method was examined using a concentration range that encompassed 8–125% of the target sample concentration for pyriproxyfen, 4–120% for permethrin and 16–110% for alpha-cypermethrin. As presented in Table 2, a linear relationship was obtained between peak area and total concentration of permethrin, alpha-cypermethrin and pyriproxyfen with regression coefficient values close to 1.0 (R^2^ > 0.9994). For all tested insecticides, the Y intercepts were effectively zero. The slope agreement was ≤ 5.8% relative standard deviation (%RSD) for permethrin, ≤ 2.2% for alpha-cypermethrin and ≤ 0.28% for pyriproxyfen.

**Table 2:** Linearity parameters, regression equations, correlation coefficients (R^2^), and standard deviations (SD) found during linearity, LoQ, and LoD testing*.

Insecticide	Amount interval	Equation	R^2^	Slope ± SD	%RSD
Permethrin^a^ (*trans* + *cis*)	31.25–1000 µg/(0.625–20 µg)	Y = 1.0517X + 8.9	0.9996	1.0517 ± 0.007	0.66
Permethrin^b^ (*trans* + *cis*)	0.24–250 µg/(4.8 ng–5 ug)	Y = 0.9938X − 0.4	0.9994	0.9938 ± 0.06	5.8
Alpha-cypermethrin^a^	31.25–500 µg/(0.625–10 µg)	Y = 1.0384X − 5.8	0.9994	1.0384 ± 0.0004	0.04
Alpha-cypermethrin^b^	0.244–250 µg/(4.8 ng–5 ug)	Y = 1.056733X + 0.5	0.9996	1.056 ± 0.02	2.2
Pyriproxyfen^a^	31.25–500 µg/(0.625–10 µg)	Y = 1.087X + 3.3	0.9999	1.087 ± 0.003	0.28
Pyriproxyfen^b^	0.03–500 µg/(0.61 ng–10 ug)	Y = 1.114X + 0.2	0.9999	1.114 ± 0.0125	0.13

*Chromatographic conditions used: 70% acetonitrile: 30% water isocratic mobile phase, 1/min flow rate, 40-min run time and an analysis wavelength of 226 nm. The column used for analysis was a Hypersil GOLD C18 column (75 Å, 250 × 4.6 mm, 5-μm particle size; Thermo Scientific). ^a^Data obtained from linearity validation where ^b^data obtained from LoQ and LoD calculation. A triplicate set of standards were prepared for each insecticide. SD; standard deviation and % RSD; relative standard deviation (SD/Mean*100).

### Accuracy and precision

The insecticide recoveries from blank nets fortified with known quantities of insecticide are presented in Table 3. Permethrin recovery ranged from 101 to 111%, alpha-cypermethrin recovery ranged from 97.7 to 99.4%, while pyriproxyfen recovery ranged from 105 to 107%. The %RSD was 0.8% for both pyriproxyfen and *alpha*-cypermethrin and 3.8 for permethrin. Thus, the insecticide recovery for all insecticides examined was close to actual values with high precision.

**Table 3:** Accuracy and precision test for blank net fortified with permethrin, alpha-cypermethrin and pyriproxyfen active ingredients.

Sample replicate	[Permethrin]		[Alpha-cypermethrin]		[Pyriproxyfen]	
	(g/kg)	Recovery %	(g/kg)	Recovery %	(g/kg)	Recovery %
1	20.3	101.5	5.362499	98.1	10.6	105.7
2	20.9	104.4	5.384918	97.9	10.7	107.1
3	21.0	105.1	5.46651	99.4	10.7	107.4
4	22.2	111.1	5.374063	97.7	10.6	106.0
Mean ± SD	21.1 ± 0.8	105.5 ± 4.0	5.4 ± 0.04	98.3 ± 0.76	10.7 ± 0.1	106.6 ± 0.8
%RSD	3.8	3.8	0.8	0.8	0.8	0.8

*SD* standard deviation and *% RSD* relative standard deviation (SD/Mean*100).

### Heat stability

Given the chiral properties of pyrethroids and pyriproxyfen (Fig. 1) and the known vulnerability of pyrethroids to degrade or isomerize upon exposure to light, heat, and solvents^16,17^, the three insecticides were assessed for their heat stability and resistance to isomerization during extraction. The stability data for permethrin, alpha-cypermethrin and pyriproxyfen before and after heating at 85 °C for 45 min are presented in Table 4. The corresponding HPLC chromatograms are shown in Figs. S3, S4 and S5 for permethrin, alpha-cypermethrin and pyriproxyfen, respectively. The quantity of the heated standards (permethrin, alpha*-*cypermethrin and pyriproxyfen) was equal to the unheated standards (Table 4). None of the examined insecticides demonstrated any signs of degradation/isomerization under the conditions tested (Figs S3, S4 and S5).

**Table 4:** Stability of permethrin and pyriproxyfen active ingredients heated at 85 °C for 45 min.

Treatment	Insecticide RT		n	[Insecticide] mg/ ± SD	%RSD
Permethrin	*Trans*	*Cis*			
0.2 mg/(Heated)	25.5	29.6	3	0.207 ± 0.00016	0.08
0.2 mg/(Unheated)	25.46 ± 0.06	29.5	3	0.202 ± 0.00002	0.01
0.4 mg/(Heated)	25.5	29.56 ± 0.06	3	0.405 ± 0.00028	0.06
0.4 mg/(Unheated)	25.5 ± 0.06	29.63 ± 0.06	3	0.399 ± 0.00032	0.08
**Alpha-cypermethrin**					
0.2 mg/(heated)	21.63 ± 0.03		3	0.19 ± 4.2E-05	0.04
0.2 mg/(Unheated)	21.65 ± 0.05		3	0.19 ± 2.7E-05	0.04
0.4 mg/(Heated)	21.61 ± 0.02		3	0.41 ± 0.001	0.8
0.4 mg/(Unheated)	21.61 ± 0.06		3	0.41 ± 0.0003	0.33
**Pyriproxyfen**					
0.2 mg/(Heated)	11.6 ± 0.0		3	0.19 ± 0.0002	0.12
0.2 mg/(Unheated)	11.63 ± 0.05		3	0.19 ± 0.0001	0.04
0.4 mg/(Heated)	11.6 ± 0.0		3	0.40 ± 0.0032	0.8
0.4 mg/(Unheated)	11.56 ± 0.06		3	0.39 ± 0.0013	0.33

*RT* insecticide peak retention time, *n* the number of replicates, *SD* Standard deviation, *%RSD* relative standard deviation (S.D./Mean*100).

### Analysis of the total active ingredient(s) content from polyethylene-based LLIN formulations

A range of LLIN formulations (Table 1) were used to evaluate the optimized method as a QCA method for insecticide(s) incorporated into polyethylene-based LLIN formulations and to validate the method reproducibility.

### Analysis of LLINs that incorporate a single insecticide

**Figure 4:**
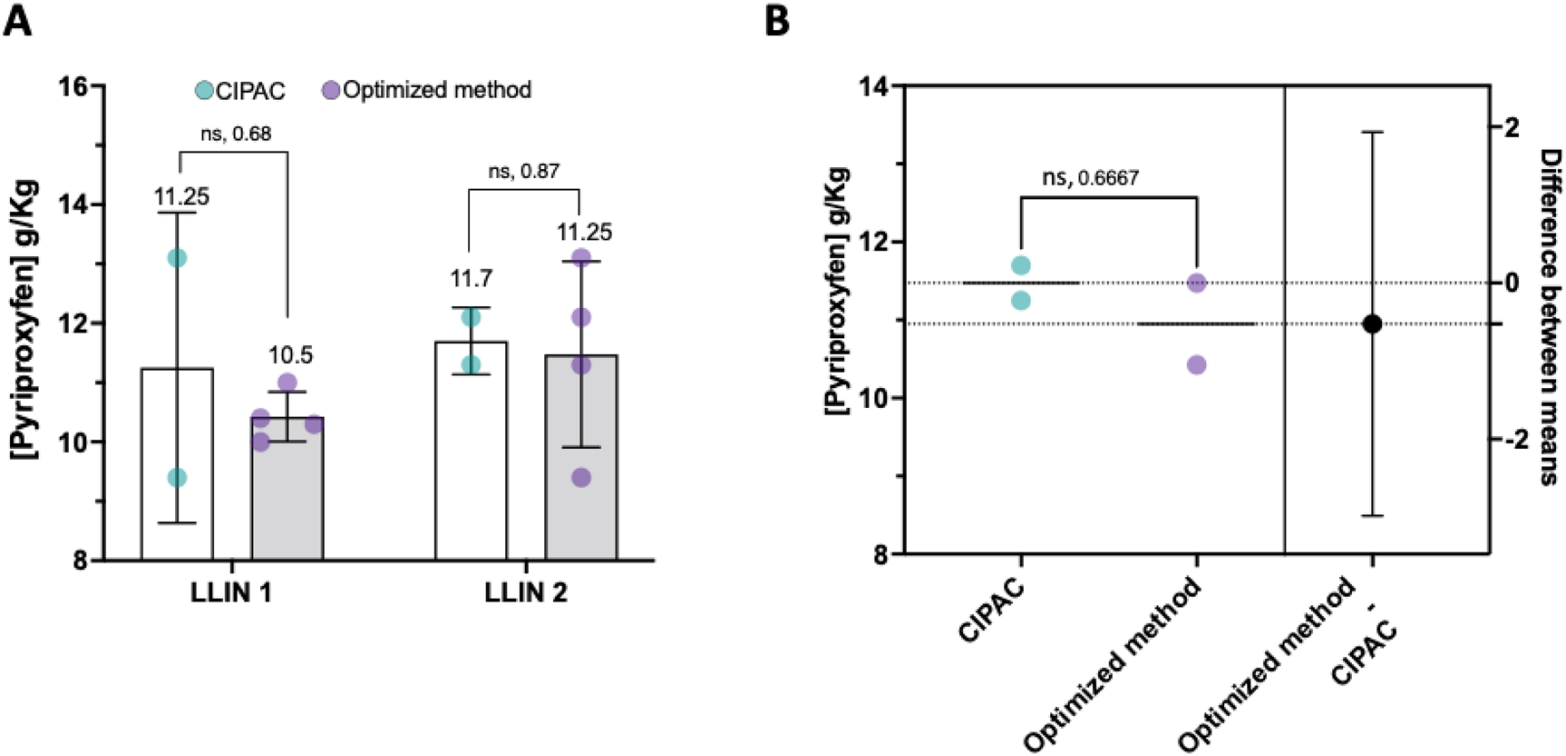
Comparison of pyriproxyfen content in prototype pyriproxyfen-treated LLIN by standard CIPAC and optimized method. (**A**) Quantity of pyriproxyfen recovered from pyriproxyfen-Net by standard CIPAC protocol vs optimized method. Multiple comparison tests were used to compare the significance of variation between the pyriproxyfen content estimated by the two methods for each LLIN. (**B**) The magnitude of difference between the optimized method and established CIPAC protocol (0.5250 ± 0.5712) with 95% CI (-2.983 to 1.933). An unpaired *t-test* was used to calculate the significant difference between the two methods at the *p*-value of 0.67. ns; no significance.

Firstly, to investigate the agreement between the optimized method and CIPAC protocol for the analysis of pyriproxyfen content, a prototype net produced by Sumitomo (Table 1) was analyzed by the optimized method and compared with the standard CIPAC protocol for QCA of pyriproxyfen content in LLIN^13^. Samples were analyzed in duplicate as recommended by the standard CIPAC protocol^13^ and in quadruplet by the new method to account for possible variability in insecticide quantities due to mosaic distribution of a.i. in net material. Graphs comparing data obtained from the two protocols are presented in Fig. 4. The CIPAC method detected 11.25 and 11.7 g/kg for LLIN1 and 2 respectively versus 10.5 and 11.25 g/ kg for the optimized method, which matched the manufactuers target dose 10 ± 2.5 g/Kg. There was no significant difference in the average amount of pyriproxyfen extracted from the two nets by either method (*P* values of 0.68 and 0.87 for LLIN1 and LLIN2 (Fig. 4A) with differences between the two methods close to zero (Fig. 4B).

**Figure 5:**
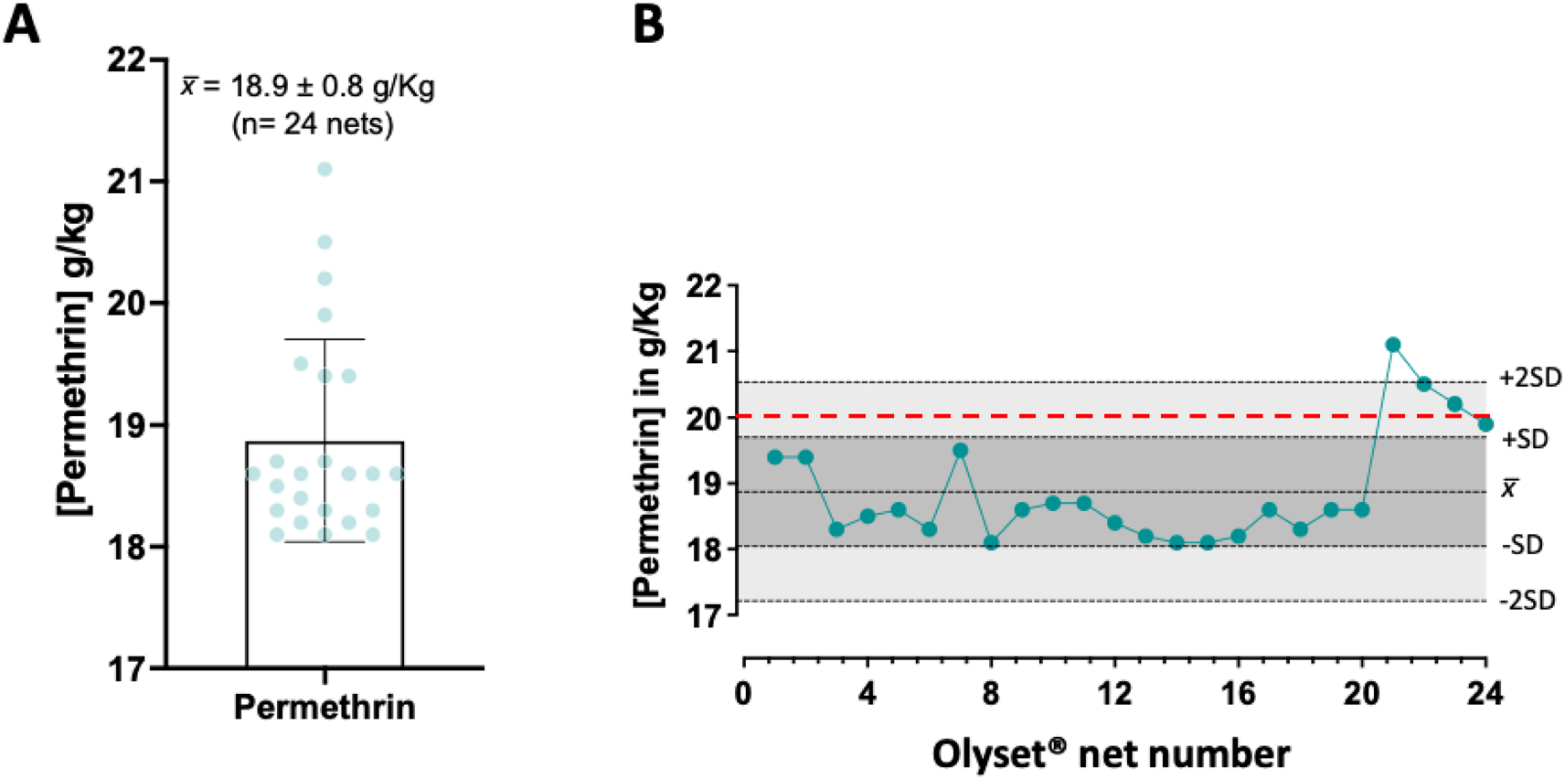
Analysis of total permethrin content in Olyset® net. (**A**) Permethrin ± standard deviation (SD) for 24 nets analyzed by the optimized method. (**B**) Levy-Jenning's chart for pyriproxyfen content in 24 LLINs was analyzed in triplicate (72 samples in total) by the optimized method. An average (x̄) of 18.9 ± 0.8 g permethrin/kg (w/w) determined for Olyset® Net (n = 24) in reference to the target concentration of 20 g/kg as declared by the manufacturer and indicated as a dotted red line on the graph.

Next, we assessed the utility of the optimised method to quantify permethrin in Olyset® net, a representative set of standard manufactured LLIN recommended by WHOPES (currently known as PQT-VC) that are incorporated with permethrin at a target dose of 20 g/kg permethrin (2% w/w). To estimate method roubstness and reproducibility for analysis of permethrin content a 24 Olyset® nets were analysed in triplicate. Consistent with WHOPES recommendations^11^, none of the 24 nets scored an average content that differed from that declared by the manufacturer by more than ± 25% (Fig. 5A). Additionally, the method presented a satisfactory level of robustness and reproducibility, as indicated from QCA data shown in Fig. 5B. Out of 24 nets, 23 scored values within ± 2SD of the 18.9 g/kg average while the 21.1 g/kg outlier remains within the WHOPES recommended range 20 ± 5 g/kg. The relative standard deviation (%RSD) of permethrin content was < 10% for all 24 nets analyzed in triplicate (Table S1), demonstrating the high precession and reproducibility of the HPLC method for permethrin quantification.

### Analysis of LLINs that incorporate two active ingredients

**Figure 6:**
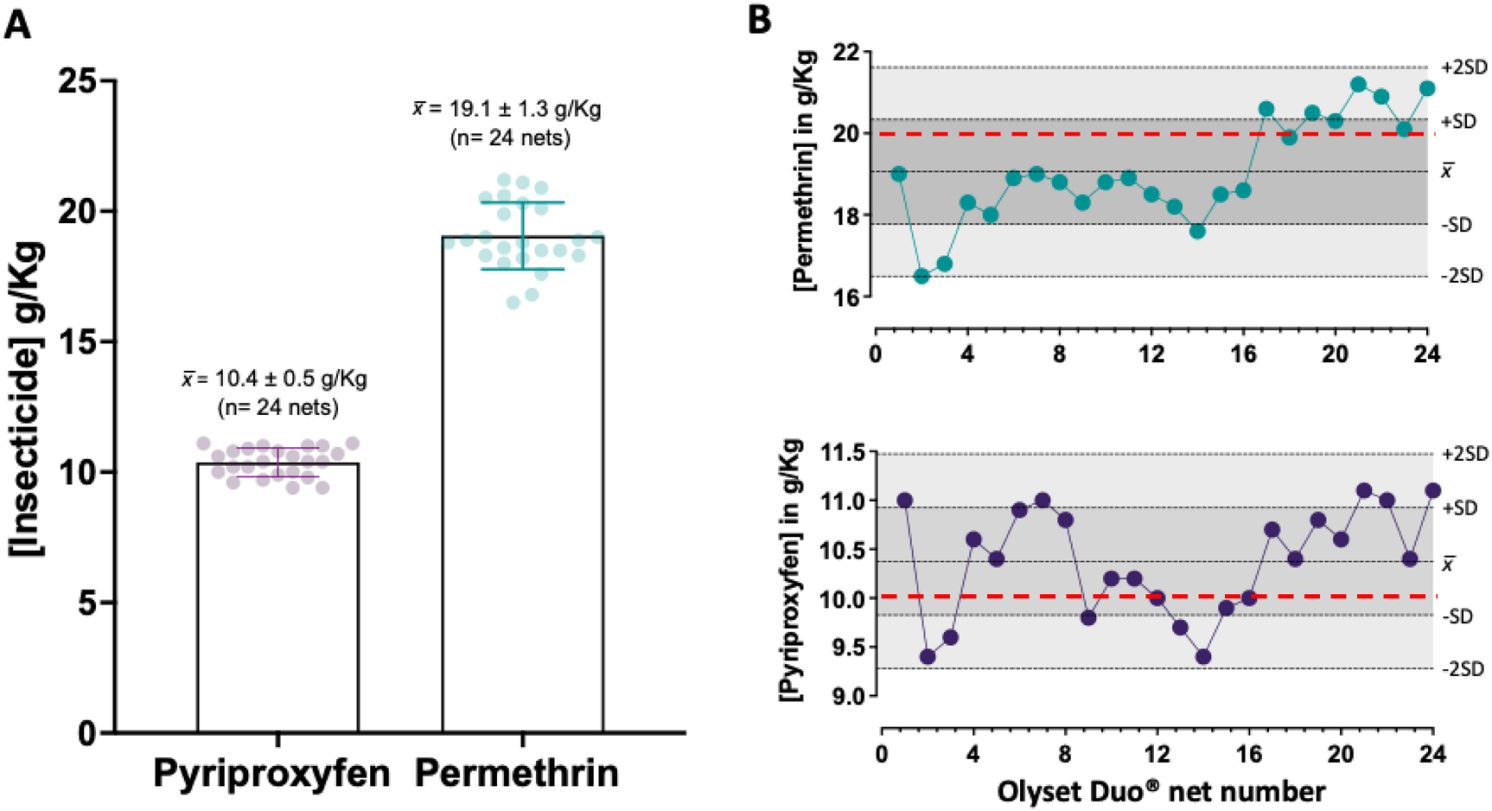
Analysis of total pyriproxyfen and permethrin content in Olyset® Duo LLIN. (**A**) The optimised method analysed the average content of pyriproxyfen and permethrin ± standard deviation (SD) for 24 Olyset® Duo. (**B**) Levy-Jenning's chart for the 24 nets analyzed in triplicates (n = 72 samples) by the optimized method. Pyriproxyfen (top chart) and permethrin (bottom chart) scored an average (x̄) of 10 ± 0.5 and 19.1 ± 1.3 g/kg, respectively. Reference concentrations for both active ingredients declared by the manufacture are denoted as red dotted lines on the charts.

Twenty four new Olyset® Duo (2% permethrin and 1% pyriproxyfen) were investigated for the simultaneous measurement of pyriproxyfen and permethrin content in LLIN polyethylene polymer following the optimized protocol. The Olyset® Duo (Sumitomo Chemical Co. Ltd.) is a prototype net containing the pyrethroid permethrin plus pyriproxyfen that is shown to kill pyrethroid-resistant *An. gambiae* mosquitoes and sterilize surviving blood-feeding mosquitoes^8,18^. None of the 24 nets scored an average dual insecticide content that differed from the amount declared by the manufacture by more than ± 25% (Fig. 6A). The method showed high accuracy and precision, as indicated by QCA data (Fig. 6B and Table S2). All nets scored values within ± 2SD of the average of 19.1 ± 1.3 g/kg for permethrin and 10.4 ± 0.5 g/kg for pyriproxyfen (Fig. 6B). An indicative of the high precision of the HPLC method, the %RSD of permethrin and pyriproxyfen content obtained from all samples analyzed in triplicate was less than 10% (Table S2).

### Royal Guard® net

**Figure 7:**
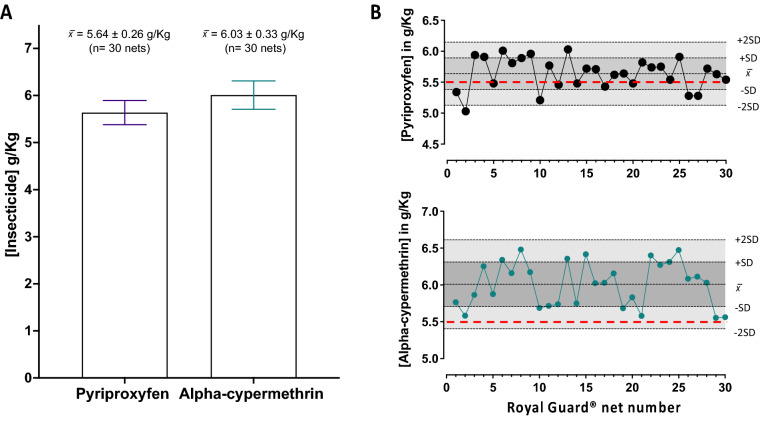
Analysis of total pyriproxyfen and alpha-cypermethrin content in Royal Guard® LLIN. (**A**) The average content of pyriproxyfen and alpha-cypermethrin ± standard deviation (SD) for 30 Royal Guard® nets. (**B**) Levy-Jenning's charts for the 30 nets were analyzed by the optimized method. Pyriproxyfen (top chart) and alpha-cypermethrin (bottom chart) scored an average (x̄) of 5.64 ± 0.26 and 6.03 ± 0.33 g/kg, respectively. Reference concentrations for both active ingredients declared by the manufacture are denoted as red dotted lines on the charts.

To establish a broader applicability of the new method for next-generation LLINs that are commercially available for malaria control, thirty Royal Guard® Nets were assessed for insecticides content. None of the 30 nets scored an insecticide content that differed from the declared manufacturer's 5.5 g/kg concentration by more than ± 25% (Fig. 7). However, a slight increase in the alpha-cypermethrin content has been noted, giving a value of 6.03 ± 0.33 g/kg (Fig. 7B).

The manufactured loading of active ingredient contents was further investigated by taking a random net from the 30 nets and subjecting it to five cycles of insecticide extraction in triplicate. The majority of the active ingredients were extracted in the first run (Fig. 6). Pyriproxyfen quantity recovered in the first round of the extraction was 5.4 ± 0.46 g/kg and alpha-cypermethrin quantity was 5.6 ± 0.14 g/kg, which is approximately equivalent to the manufacturer’s reference value for both insecticides (5.5 ± 1.375 g/kg) (Fig. 6). Compared to the first run, a negligible amount of the two active ingredients were recovered in the subsequent four runs, accounting to a residual amount of 0.02 and 0.6 g/kg of pyriproxyfen and alpha-cypermethrin likely carried over from the first run (Fig. 6).

The accuracy and precision of the method for QCA of Royal Guard**®** net was evaluated by intraday and interday analysis. The relative standard deviation of both intraday and interday precision was ≤ 3.4% (Table 5). Moreover, pyriproxyfen and alpha-cypermethrin recovery were estimated at 106.9 and 94.3%, respectively, from the same quality control samples (Table 5).

**Table 5:** Precision and accuracy of alpha-cypermethrin and pyriproxyfen extracted from Royal Guard® LLIN.

Insecticide	Target Concentration (g/Kg)	Accuracy (% nominal)	Precision (%RSD)	
			Intraday (n = 6)	Interday (n = 18)
Alpha-Cypermethrin	5.5	94.3	2.24	3.54
Pyriproxyfen	5.5	106.9	2.93	2.6

## Discussion

We have developed a simplified approach for sample preparation, extraction and insecticide quantification from LLINs made from polyethylene polymers that incorporate pyrethroid and pyriproxyfen insecticides. The standard CIPAC protocol for the QCA of pyriproxyfen net recommends heating large amounts of net material (~ 2 g) with 50 of the solvent mixture at 85–90 °C in duplicate, which results in the production of a significant amount of solvent waste that if scaled for multiple nets could be problematic for public health and the environment^15,19,20^. Solvent selection guideline has identified heptane as a problematic but not hazardous solvent^15,20^. By reducing the sample size to ~ 0.2 g we were able to reduce the solvent used for extraction by tenfold, providing greener chemistry and sustainable solvent use in chemical processing, and eliminating the need for rotary evaporation that prevents the facile evaporation of multiple samples for high throughput analysis of multiple LLINs. Chromatographic conditions were also optimized for the separation and quantitation of pyriproxyfen, permethrin and alpha-cypermethrin. The U.V. detection wavelength of 226 nm and mobile phase composition of 70% acetonitrile in water has helped to achieve higher sensitivity for insecticide detection and quantification with the small sample size (0.2 g) at shorter 30–40 min run time relative to CIPAC (60 min)^13^.

The extraction and recovery of additives incorporated into a plastic polymer can be also difficult and usually requires the complete dissociation and solvation of the polymer material using hazardous solvents such as xylene at high temperature (> 140 °C). With our protocol, heating LLINs with heptane at 85 °C for 45 min was sufficient to recover insecticides (permethrin, alpha-cypermethrin and pyriproxyfen) from the polyethylene fibers by swelling of the polymer without dissolving the fibre. Similarly, iso-octane has been tested previously as a universal solvent for pyrethroid extraction from polyester and polyethylene nets without dissolving fibre^14^. However, the extraction was reliant on large sample size and lacked an internal standard^14^, thus prone to variability in insecticide quantification due to solvent volatility. In contrast, our method doesn't preclude the internal standard (DCP) recommended in the original CIPAC protocol^13^, resulting in a more robust and reproducible method for the quantitative analysis of the active ingredients from LLINs (Figs. 5, 6and 7).

The new method facilitates the analysis of insecticides by enabling multiple net samples to be processed in parallel using standard low volume tubes and multiwall dry blocks for solvent evaporation (Fig. 2). Coupled with the higher-sensitivity of HPLC and shorter run times, this greatly speeds up the processing and data collection to analyze LLIN insecticide content. In our hands, one operator can run up to 40 LLINs in triplicate per HPLC run. Moreover, the stability of the insecticides has not been altered during the extraction process as indicated from heat stability data (Table 4) which should result in no alteration of their biological activity. Collectively this qualifies our protocol to be used for quality control purposes to measure pyriproxyfen and pyrethroid content incorporated in LLINs as demonstrated by the use of the method in field trials in Burkina Faso and Benin that tested the efficacy of Olyset® Duo LLIN^8,18^. Here, the optimised method has been further refined and evaluated for linearity, specificity, accuracy and precision and found suitable for insecticide quantification from various types of LLINs that incorporate pyriproxyfen, permethrin and alpha-cypermethrin. These include the commercially available Olyset® Net that contains permethrin and has been used extensively for malaria control operations in Africa and Royal Guard® Net a new LLIN that contains a mixture of alpha-cypermethrin and pyriproxyfen and whose use is likely to escalate in future^10^.

The optimised method, which allows the scale-up of insecticide extraction from LLINs offers a relatively simple and cost effective means of performing analytical checks for QCA purposes that would be accessible for most laboratories. Moreover, we anticipate that our method will be valid for other prequalified approved ITNs by PQT-VC (Supplementary data 1) contain pyrethroid insecticides and is the subject of future research.

## Supplementary Information

Supplementary Information.

Supplementary Data.

## References

[1] WHO. World malaria report. *Geneva. Switzerland,* (2018).

[2] Bhatt S (2015). The effect of malaria control on Plasmodium falciparum in Africa between 2000 and 2015. Nature.

[3] WHO. World malaria report. *Geneva. Switzerland,* (2020).

[4] Zaim M, Aitio A, Nakashima N (2000). Safety of pyrethroid-treated mosquito nets. Med. Vet. Entomol..

[5] Strode C, Donegan S, Garner P, Enayati AA, Hemingway J (2014). The impact of pyrethroid resistance on the efficacy of insecticide-treated bed nets against African anopheline mosquitoes: systematic review and meta-analysis. PLoS Med..

[6] Ranson H (2011). Pyrethroid resistance in African anopheline mosquitoes: what are the implications for malaria control?. Trends Parasitol..

[7] Denholm I, Rowland MW (1992). Tactics for managing pesticide resistance in arthropods: theory and practice. Annu. Rev. Entomol..

[8] Ngufor C (2016). Efficacy of the Olyset Duo net against insecticide-resistant mosquito vectors of malaria. Sci. Transl. Med..

[9] Mosha JF (2021). Protocol for a four parallel-arm, single-blind, cluster-randomised trial to assess the effectiveness of three types of dual active ingredient treated nets compared to pyrethroid-only long-lasting insecticidal nets to prevent malaria transmitted by pyrethroid insecticide-resistant vector mosquitoes in Tanzania. BMJ Open.

[10] Ngufor C, Agbevo A, Fagbohoun J, Fongnikin A, Rowland M (2020). Efficacy of Royal Guard, a new alpha-cypermethrin and pyriproxyfen treated mosquito net, against pyrethroid-resistant malaria vectors. Sci. Rep..

[11] WHO. Guidelines for laboratory and field-testing of long-lasting insecticidal nets *World Health Organization* (2013).

[12] CIPAC. Permethrin long lasting insecticidal net 331/LN/M. *Collaborative International Pesticides Analytical Council* Handbook M, 158 (2007).

[13] CIPAC. Pyriproxyfen long lasting insecticidal net *715/LN/M/. *Collaborative International Pesticides Analytical Council* Handbook O, 143 (2014).

[14] Jenkins DW (2013). Development and validation of a 'universal' HPLC method for pyrethroid quantification in long-lasting insecticidal mosquito nets for malaria control and prevention. Trop. Med. Int. Health.

[15] Welton T (2015). Solvents and sustainable chemistry. Proc. Math. Phys. Eng. Sci..

[16] Liu W, Qin S, Gan J (2005). Chiral stability of synthetic pyrethroid insecticides. J. Agric. Food Chem..

[17] Harburguer LV (2009). Thermal behaviour and biological activity against Aedes aegypti (Diptera: Culicidae) of permethrin and pyriproxyfen in a smoke-generating formulation. Pest. Manag. Sci..

[18] Toe KH (2019). Assessing the impact of the addition of pyriproxyfen on the durability of permethrin-treated bed nets in Burkina Faso: a compound-randomized controlled trial. Malar. J..

[19] Clarke CJ, Tu WC, Levers O, Brohl A, Hallett JP (2018). Green and sustainable solvents in chemical processes. Chem. Rev..

[20] Prat D, Hayler J, Wells A (2014). A survey of solvent selection guides. Green Chem..

