# Supplementary material for: A high-throughput HPLC method for simultaneous quantification of pyrethroid and pyriproxyfen in long-lasting insecticide-treated nets
Source: Sci Rep. 2022 Jun 11;12:9715. doi: 10.1038/s41598-022-13768-z (PMC9188574; doi:10.1038/s41598-022-13768-z)
Supplement: Supplementary file 1 — Supplementary Information. [file 41598_2022_13768_MOESM1_ESM.docx]

## **Supplementary Information**

**A high-throughput HPLC method for simultaneous quantification of pyrethroid and pyriproxyfen in long-lasting insecticide-treated nets**

Kyle J. Walker^1*^, Christopher T. Williams^1*^, Folasade O. Oladepo^1^, John Lucas^2^, David Malone^3^, Mark J.I. Paine^1^, and Hanafy M. Ismail^1§^

^1^Vector Biology Department, Liverpool School of Tropical Medicine, Pembroke Pl, Liverpool L3 5QA, U.K.

^2^John Richard Lucas, Technical Consultant, Cowleigh Park Farm, Cowleigh Road, Malvern

WR13 5HJ, U.K.

^3^Innovative Vector Control Consortium, Liverpool School of Tropical Medicine, Pembroke Place, Liverpool L3 5QA, U.K.

# ^*^Shared first authorship

^§^Contact: [Hanafy.ismail@lstmed.ac.uk](mailto:Hanafy.ismail@lstmed.ac.uk)


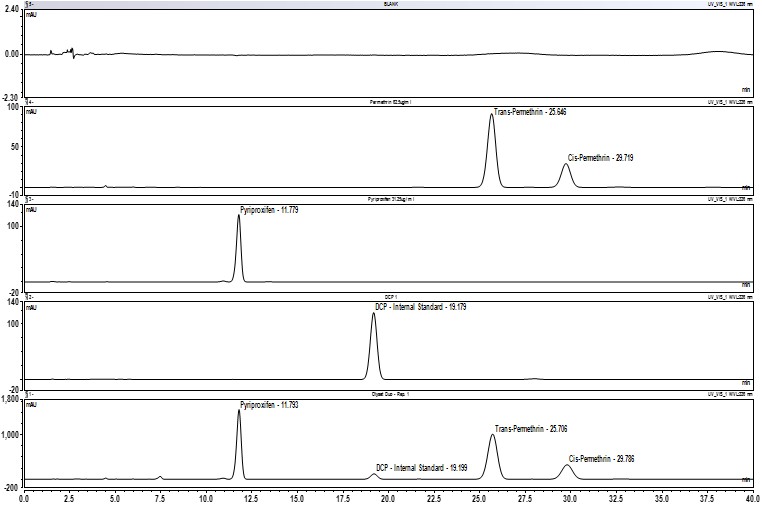


**Figure S1**. HPLC chromatograms for insecticides extracted from Olyset^®^ Duo and analytical standards insecticide. From top to bottom: blank sample solution, permethrin analytical standard, pyriproxyfen analytical standard, internal standard ‘dicyclohexyl phthalate (DCP)’, and sample solution following extraction of Olyset^®^ Duo. The calculated percentage retention time (%RT= RT _sample_/RT _std_ x 100) found 100.11 (pyriproxyfen), 100.1 (DCP), 100.23 (trans-permethrin), 100.22 (c*is*-permethrin). Peaks purity in sample solution was 997 for pyriproxyfen, 1000 for trans-permethrin and 1000 for *cis*-permethrin.

**Figure S2.** HPLC chromatograms for insecticides extracted from Royal Guard^®^ Net and analytical standards insecticide. From top to bottom: blank sample solution, alpha-cypermethrin analytical standard, pyriproxyfen analytical standard, internal standard ‘dicyclohexyl phthalate (DCP)’, sample solution following extraction of Royal Guard^®^ LLIN. The calculated percentage retention time (%RT= RT _sample_/RT _std_ x 100) found 99.82% (Pyriproxyfen), 99.77% (DCP), and 100.07% (alpha-cypermethrin). Peaks purity in sample solution was 997 for pyriproxyfen and 1000 for alpha-cypermethrin.

**Figure S3.** HPLC chromatograms for permethrin heat stability test. **(**A) heated vs unheated 0.2 mg/mL permethrin standards. (B) heated vs unheated 0.4 mg/mL permethrin standards.

**Figure S4.** HPLC chromatograms for alpha-cypermethrin heat stability test. (A) heated vs unheated 0.2 mg/mL alpha-cypermethrin standards. (B) heated vs unheated 0.4 mg/mL alpha-cypermethrin standards.

**Figure S5.** HPLC chromatograms for pyriproxyfen heat stability test. **(**A) heated vs unheated 0.2 mg/mL pyriproxyfen standards. (B) heated vs unheated 0.4 mg/mL pyriproxyfen standards.

**Table S1.** Analysis of permethrin content in unused Olyset^®^ nets

| **Sample name** | ***n*** | **Target concentration (g/Kg)** | **Permethrin g/Kg ± SD** | **Precision (%RSD)** | **Accuracy^§^ (% nominal)** |
| --- | --- | --- | --- | --- | --- |
| Olyset 1 | 3 | 20 | 19.4 ± 1 | 5.2 | 97 |
| Olyset 2 | 3 | 20 | 19.4 ± 0.2 | 1.2 | 97 |
| Olyset 3 | 3 | 20 | 18.3 ± 0.3 | 1.8 | 91.5 |
| Olyset 4 | 3 | 20 | 18.5 ± 0.1 | 0.5 | 92.5 |
| Olyset 5 | 3 | 20 | 18.6 ± 0.8 | 4.2 | 93 |
| Olyset 6 | 3 | 20 | 18.3 ± 0.1 | 0.7 | 91.5 |
| Olyset 7 | 3 | 20 | 19.5 ±1.8 | 9.1 | 97.5 |
| Olyset 8 | 3 | 20 | 18.1 ± 0.1 | 0.5 | 90.5 |
| Olyset 9 | 3 | 20 | 18.6 ± 0.2 | 1 | 93 |
| Olyset 10 | 3 | 20 | 18.7 ± 0.1 | 0.4 | 93.5 |
| Olyset 11 | 3 | 20 | 18.7 ± 0.3 | 1.5 | 93.5 |
| Olyset 12 | 3 | 20 | 18.4 ± 0.2 | 1.3 | 92 |
| Olyset 13 | 3 | 20 | 18.2 ± 0.0 | 0.2 | 91 |
| Olyset 14 | 3 | 20 | 18.1± 0.1 | 0.7 | 90.5 |
| Olyset 15 | 3 | 20 | 18.1 ± 0.1 | 0.7 | 90.5 |
| Olyset 16 | 3 | 20 | 18.2 ± 0.1 | 0.7 | 91 |
| Olyset 17 | 3 | 20 | 18.6 ± 0.1 | 0.7 | 93 |
| Olyset 18 | 3 | 20 | 18.3 ± 0.3 | 1.5 | 91.5 |
| Olyset 19 | 3 | 20 | 18.6 ± 0.3 | 1.5 | 93 |
| Olyset 20 | 3 | 20 | 18.6 ± 0.2 | 1.2 | 93 |
| Olyset 21 | 3 | 20 | 21.1 ± 0.4 | 1.7 | 105.5 |
| Olyset 22 | 3 | 20 | 20.5 ± 0.2 | 1 | 102.5 |
| Olyset 23 | 3 | 20 | 20.2 ± 0.2 | 1.1 | 101 |
| Olyset 24 | 3 | 20 | 19.9 ± 0.1 | 0.6 | 99.5 |

n; number of replicates, S.D.; standard deviation (n=3), %RSD: relative standard deviation (S.D./Mean*100).

^§^ Accuracy = (mean concentration found/target concentration) ×100

**Table S2.** Analysis of permethrin and pyriproxyfen content in unused Olyset^®^ Duo LLIN

| **Sample name** | ***n*** | **Pyriproxyfen g/kg ± SD** | **Precision (%RSD)** | **Accuracy^§^** | **Permethrin g/kg ± SD** | **Precision (%RSD)** | **Accuracy^§^** |
| --- | --- | --- | --- | --- | --- | --- | --- |
| Olyset Duo 1 | 3 | 11 ± 0.3 | 2.4 | 110 | 19 ± 0.4 | 2.2 | 95 |
| Olyset Duo 2 | 3 | 9.4 ± 0.2 | 2.4 | 94 | 16.5 ± 0.4 | 2.1 | 82.5 |
| Olyset Duo 3 | 3 | 9.6 ± 0.1 | 0.7 | 96 | 16.8 ± 0.1 | 0.6 | 84 |
| Olyset Duo 4 | 3 | 10.6 ± 0 | 0.1 | 106 | 18.3 ± 0.0 | 0.2 | 91.5 |
| Olyset Duo 5 | 3 | 10.4 ± 0 | 0.2 | 104 | 18 ± 0.0 | 0.08 | 90 |
| Olyset Duo 6 | 3 | 10.9 ± 0.4 | 3.3 | 109 | 18.9 ± 0.6 | 3.3 | 94.5 |
| Olyset Duo 7 | 3 | 11 ± 0.3 | 2.3 | 110 | 19 ± 0.4 | 2.06 | 95 |
| Olyset Duo 8 | 3 | 10.8 ± 0.2 | 2.2 | 108 | 18.8 ± 0.4 | 1.9 | 94 |
| Olyset Duo 9 | 3 | 9.8 ± 0.4 | 4.3 | 98 | 18.3 ± 0.8 | 4.3 | 91.5 |
| Olyset Duo 10 | 3 | 10.2 ± 0.2 | 1.6 | 102 | 18.8 ± 0.3 | 1.5 | 94 |
| Olyset Duo 11 | 3 | 10.2 ± 0.2 | 2 | 102 | 18.9 ± 0.4 | 2.0 | 94.5 |
| Olyset Duo 12 | 3 | 10 ± 0.1 | 0.5 | 100 | 18.5 ± 0.1 | 0.5 | 92.5 |
| Olyset Duo 13 | 3 | 9.7 ± 0.1 | 1.3 | 97 | 18.2 ± 0.3 | 1.4 | 91 |
| Olyset Duo 14 | 3 | 9.4 ± 0.6 | 6.1 | 94 | 17.6 ± 0.9 | 5.3 | 88 |
| Olyset Duo 15 | 3 | 9.9 ± 0.2 | 1.6 | 99 | 18.5 ± 0.3 | 1.6 | 92.5 |
| Olyset Duo 16 | 3 | 10 ± 0.0 | 0.5 | 100 | 18.6 ± 0.0 | 0.2 | 93 |
| Olyset Duo 17 | 3 | 10.7 ± 0.2 | 1.5 | 107 | 20.6 ± 0.3 | 1.3 | 103 |
| Olyset Duo 18 | 3 | 10.4 ± 0.1 | 0.8 | 104 | 19.9 ± 0.2 | 0.8 | 99.5 |
| Olyset Duo 19 | 3 | 10.8 ± 0.1 | 1.2 | 108 | 20.5 ± 0.3 | 1.3 | 102.5 |
| Olyset Duo 20 | 3 | 10.6 ± 0 | 0.5 | 106 | 20.3 ± 0.1 | 0.4 | 101.5 |
| Olyset Duo 21 | 3 | 11.1 ± 0.2 | 1.5 | 111 | 21.2 ± 0.3 | 1.3 | 106 |
| Olyset Duo 22 | 3 | 11 ± 0.1 | 0.5 | 110 | 20.9 ± 0.1 | 0.4 | 104.5 |
| Olyset Duo 23 | 3 | 10.4 ± 0.3 | 2.7 | 104 | 20.1 ± 0.4 | 2.1 | 100.5 |
| Olyset Duo 24 | 3 | 11.1 ± 0.2 | 1.4 | 111 | 21.1 ± 0.3 | 1.2 | 105.5 |

n; the number of replicates, S.D.; standard deviation (n=3), %RSD: relative standard deviation (STDEV/Mean*100)

^§^ Accuracy = (mean concentration found/target concentration) ×100

**Figure S6.** Total insecticide content in Royal Guard^®^ net subjected to five cycles of extraction.
